# Improved bioavailability of buccal nanoemulsion vitamin D compared to conventional oral supplementation in patients with inflammatory bowel disease: a randomized controlled trial

**DOI:** 10.3389/fmed.2025.1649677

**Published:** 2025-08-25

**Authors:** Vladimír Kojecký, Josef Klhůfek, Bohuslav Kianička, Pavel Kohout

**Affiliations:** ^1^Department of Internal Medicine, Tomas Bata Hospital Zlín, Zlín, Czechia; ^2^2nd Department of Internal Medicine, St. Anne’s Faculty Hospital, Brno and Faculty of Medicine, Masaryk University, Brno, Czechia; ^3^Department of Clinical Pharmacy, Tomas Bata Hospital Zlín, Zlín, Czechia; ^4^Internal Clinic, 3rd Medical Faculty, Charles University and University Thomayer Hospital, Prague, Czechia

**Keywords:** inflammatory bowel disease, vitamin D supplementation, vitamin D bioavailability, oral absorption, nanoemulsion

## Abstract

**Objectives:**

The absorption of conventional cholecalciferol may be impaired in patients with inflammatory bowel disease (IBD). The bioavailability and optimal dosing of buccally absorbable nanoemulsion vitamin D in this population remain unclear. This study aimed to compare the effects of buccal nanoemulsion and conventional oral vitamin D supplementation on serum 25-hydroxyvitamin D (25OHD) levels in patients with IBD.

**Methods:**

This was an open-label randomized trial. Patients with IBD were assigned to receive cholecalciferol in an oil emulsion at a dose of 14 000 IU weekly (GTTS) and orally absorbed cholecalciferol at dose 4000 IU twice a week (SPRAY) for 12–16 weeks during the winter months. Plasma 25OHD levels were measured at baseline and after the supplementation period.

**Results:**

A total of 120 patients were analyzed. Among 75 subjects with CD and 45 with UC, 27% had active disease, and 24% of the Crohn’s disease patients had undergone ileocecal resection. The initial mean 25OHD level was 65.9 ± 21.0 nmol/l in the SPRAY group and 59.1 ± 27.7 nmol/l in the GTTS group. A similar increase of 9.3 ± 26.8 nmol/l (GTTS) and 9.2 ± 27.7 nmol/l (SPRAY) in 25OHD levels occurred in both groups, with similar variations. The proportion of subjects with normal and sub-normal levels following the substitution was comparable. The change in 25OHD level correlated negatively only with the baseline 25OHD level (*p* < 0.02) among all monitored variables.

**Conclusion:**

In IBD patients, the sufficient supplementation dose of the orally absorbable cholecalciferol is half that of the conventional oil emulsion (1143 IU/day vs. 2000 IU/day). Variable intestinal absorption is not a factor explaining inter-individual differences in 25OHD levels using a conventional vitamin D emulsion.

## 1 Introduction

Vitamin D (vitD) deficiency is widespread in the population (1). It is also common among patients with inflammatory bowel diseases (IBD). Even during summer, 18%–59% of patients with inflammatory bowel disease (IBD) exhibit insufficient serum vitamin D levels (50–75 nmol/L). In winter, this proportion rises to 50%–100%, with up to 75% of patients being deficient (< 50 nmol/L) (2). Vitamin D deficiency negatively impacts the course of IBD (3). Dietary intake is low (4) and insufficient to meet the body needs.

To prevent deficiency, vitD supplementation is necessary. Oral cholecalciferol in an oil preparation is commonly used. The absorption of these fat-soluble preparations varies considerably (5). In patients with IBD, particularly those with small intestinal involvement, vitamin D bioavailability may be even lower than in healthy individuals.

A so-called nanoemulsion of vitamin D has been developed, which exhibits higher absorption from the gastrointestinal tract compared to conventional form (6). There is also a formulation that is absorbed through the buccal mucosa, bypassing potential issues with gastrointestinal absorption. However, data on its effectiveness are limited. The buccal nanoemulsion spray has been studied only in healthy subjects and over short periods of several weeks (7, 8). The effectiveness of this formulation in IBD patients and the required supplementation dose for these individuals are not yet known.

The aim of the study is to compare the effectiveness of supplementation with conventional fat-soluble vitamin D with that of an orally absorbable nanoemulsion in IBD patients during the winter months.

## 2 Materials and methods

### 2.1 Study design

This was a prospective, randomized, open-label study conducted at conducted at the IBD center of Bata Regional Hospital in Zlin, Czech Republic. Adult outpatients (age 18–70 years) with a confirmed diagnosis of Crohn’s disease and ulcerative colitis were enrolled between October 2022 and January 2024.

The study was conducted in accordance with the Declaration of Helsinki and was approved by the Institutional Review Board of the Bata Regional Hospital. All study participants provided written informed consent. This trial is registered at https://clinicaltrials.gov (NCT05733117).

### 2.2 Study participants

The study was conducted between October and April, a period during which endogenous vitamin D synthesis is minimal due to limited sunlight exposure. The total duration ranged from 12 to 16 weeks. The interval between baseline and follow-up visits (minimum of 3 months) was selected to exceed four half-lives of 25-hydroxyvitamin D (25OHD). Sample size was calculated based on a non-inferiority design, assuming a power of 80% and a significance level of 0.05. A total of 56 participants per group were required to detect a difference of ± 4 nmol/L in 25OHD levels. We have anticipated a 20% drop-out rate and planned to enroll 67 patients in each arm. The participants were randomized using a software generated stratified permuted block randomization (block size 8) with stratum 25OHD 55 nmol/l and body weight 75 kg.

Exclusion criteria were as follows: conditions potentially affecting serum vitamin D levels, including renal insufficiency, liver and cholestatic diseases, malabsorption syndromes, celiac disease, treatment with anticonvulsants, pregnancy, prior gastrointestinal surgery (excluding standard ileo-cecal resection), or any other severe illness; hyperparathyroidism (parathormone > 8 pmol/L); hypercalcemia (Ca^2+^ > 2.65 mmol/L); inability to obtain valid data; and use of supplements containing vitamin D. Use of oral calcium supplements without vitamin D was not considered an exclusion criterion. Patients with highly active IBD, defined as Crohn’s Disease Activity Index (CDAI) > 220 or Partial Mayo Clinic Score (pMCS) > 6, were also excluded.

### 2.3 Intervention

Patients were randomly assigned to one of the cholecalciferol intervention groups: oral spray (SPRAY) using the Vitamin D3 Orofast^®^ Axonia, 1000 IU per spray (4000 IU twice a week), and conventional emulsion (GTTS) Vigantol gtt., Merck (14.000 IU/once a week). The dose of GTTS was based on our previous work (9), while the SPRAY was used at a half dose, based on prior evidence suggesting enhanced bioavailability of this form (6, 10). Since pharmacokinetic data show no significant difference between daily and weekly dosing, but adherence to daily intake is low (11), a twice-weekly regimen of SPRAY was chosen to improve compliance. To avoid confusion regarding dosing, a fixed schedule of two doses per week (2 × 4000 IU) was implemented. Patients were instructed to take the preparation in the morning and to avoid eating or drinking for 30 min after administration. Adherence was monitored using a patient diary, in which subjects recorded the number of applications (spray, drops).

Baseline demographic and anthropometric data were collected during the initial visit. At each visit, blood samples were taken to evaluate serum 25OHD concentration. Vitamin D levels were defined as total 25-hydroxyvitamin D (25OHD), calculated as the sum of 25-hydroxyvitamin D_2_ and 25-hydroxyvitamin D_3_, and were determined using an immunochemiluminescent assay (Architect, Abbott; cut-off > 75 nmol/L). VitD deficiency was defined as 25OHD < 50 nmol/L, insufficiency as 25OHD between 50 and 74 nmol/L, while levels between 75 and 250 nmol/L were considered normal (12). Non-adherence was defined as a deviation greater than ± 15% from the prescribed vitamin D supplementation dose. Serum calcium, phosphorus, parathyroid hormone (PTH) and C reactive protein, were evaluated at baseline and after intervention. Sensory properties of the preparation were not monitored.

### 2.4 Outcome measures

The primary outcome of the study was the change in serum 25OHD concentration from baseline to the end of the supplementation period.

### 2.5 Statistical analysis

Bivariate statistical analyses were performed using tests (*T*-test, Mann Whitney test) based on a data normality check with Shapiro-Wilk test. Categorical variables were analyzed using Pearson’s Chi-square test or Fisher’s exact test. Correlation analyses were performed using Pearson and/or Spearman’s correlation coefficients. Statistical analysis was performed using Minitab 17 software (Minitab Inc., State College, PA, USA). Continuous variables were reported as median and interquartile range, while categorical variables were expressed as absolute frequencies and percentages. *P*-values < 0.05 were considered as significant.

## 3 Results

**FIGURE 1:**
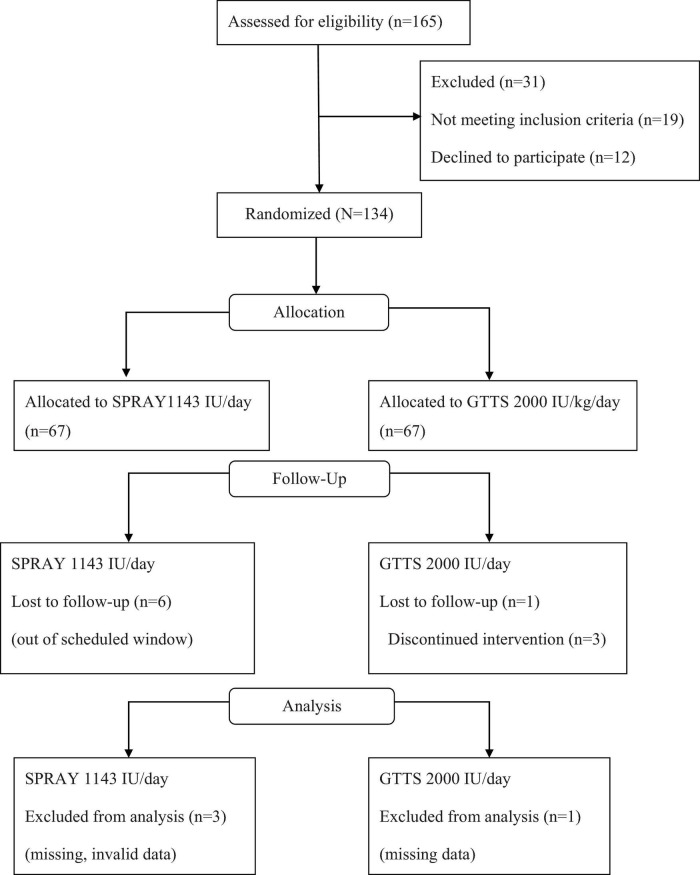
Study flowchart.

Of the 134 randomized subjects, 120 were included in the final analysis. The study flowchart is presented in Figure 1. Baseline characteristics of the two groups are summarized in Table 1. The groups were comparable in terms of age, weight, disease duration, and disease type.

**TABLE 1:** The values are presented as mean (standard deviation) or absolute frequency (percentage).

	SPRAY	GTTS	*P*-value
	(*N* = 58)	(*N* = 62)	
Gender (females)	37 (63.8%)	26 (41.9%)	0.02*
Age (years)	40.9 (18.6)	43.2 (13.9)	NS
Weight (kg)	75.8 (14.5)	76.3 (18.3)	NS
Disease duration (years)	9.5 (5.7)	8.2 (9.5)	NS
Crohn’s disease	34 (58.6%)	41 (66.1%)	NS
Ileal	13 (38.3%)	19 (46.3%)	NS
Colonic	3 (8.8%)	7 (17.1%)	NS
Ileocolonic	17 (50.0%)	12 (26.8%)	NS
Isolated upper	1 (2.9%)	3 (7.3%)	NS
Ulcerative colitis	24 (41.4%)	21 (33.9%)	NS
Left sided	14 (58.3%)	5 (23.8%)	0.03*
Pancolitis	9 (37.5%)	16 (76.2%)	0.02*
Proctitis	1 (4.2%)	0 (0.0%)	NS
Ileo-cecal resection	8 (13.8%)	10 (16.1%)	NS
Active disease	13 (22.4%)	19 (30.6%)	NS

*Significant (*p* < 0.05), NS, not significant.

At baseline, the initial mean serum 25OHD level was 65.9 ± 21.0 nmol/l in the SPRAY group and 59.1 ± 27.7 nmol/l in the GTTS group. After 12 weeks of supplementation, both groups showed a significant increase in 25OHD levels. The 25OHD level increased by 9.3 ± 26.8 nmol/l (*p* = 0.008) in the GTTS group and by 9.2 ± 27.7 nmol/l (*p* = 0.014) in the SPRAY group.

**FIGURE 2:**
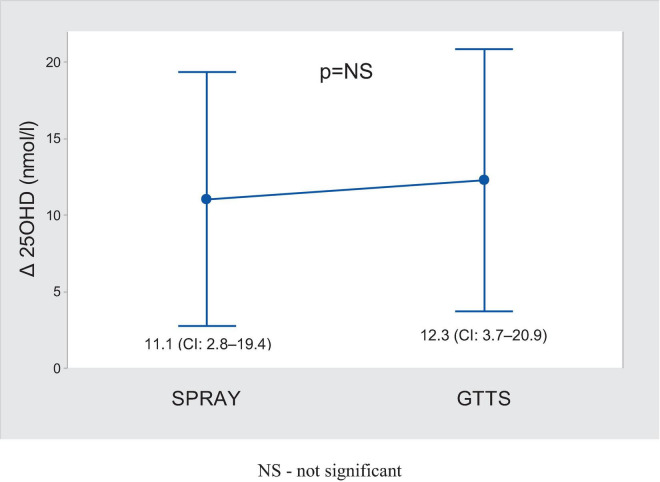
Comparison of changes in blood 25OHD after supplementation (mean ± 95% CI).

**FIGURE 3:**
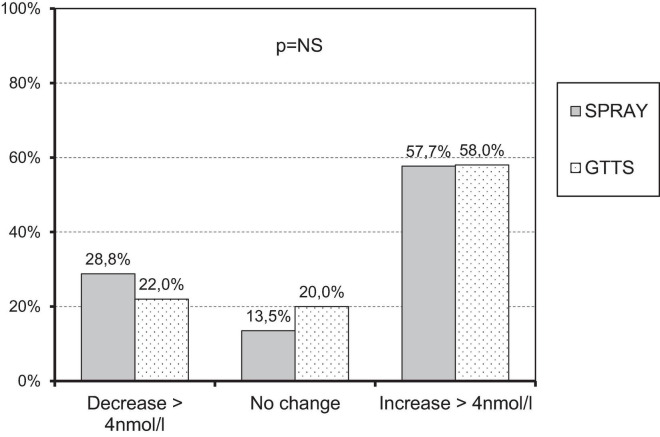
Proportion of subjects with similar changes in 25OHD levels across study groups.

There were 52 adherent subjects in the SPRAY group and 50 in the GTTS group. Further analyses were conducted on these subjects (see Table 2). The average supplementation dose was 1988 ± 91 IU/day in the GTTS group and 1148 ± 39.7 IU/day in the SPRAY group. The increase in 25OHD levels following supplementation (Figure 2) was significant and similar in both groups. The variability in changes of 25OHD levels was comparable between the two groups. The maximum 25OHD level at the end of supplementation did not exceed 117 nmol/l (SPRAY). The number of subjects with sustained 25OHD levels was comparable in both groups (13.5% vs. 20.3%, SPRAY vs. GTTS). A similar distribution was observed in the proportions of subjects with increased or decreased levels (Figure 3).

**TABLE 2:** 25-hydroxyvitamin D (25OHD) levels and other parameters at the start and end of the study (complaint subjects).

	N	Initial visit	Completion visit	Change 25OHD (nmol/l)	*P*-value
Oral SPRAY	52			11.1 (29.8)	
25OHD (nmol/l)		65.9 (21.7)	75.4 (25.0)		0.018*
Ca^2+^ mmol/l		2.3 (0.11)	2.3 (0.2)		NS
P (mmol/l)		1.1(0.2)	1.2 (0.4)		NS
Parathormone		3.1(1.4)	3.3 (1.6)		NS
Conventional GTTS	50			12.3 (28.2)	
25OHD (nmol/l)		53.0 (29.2) *	65.4 (24.2) *		0.006*
Ca^2+^ (mmol/l)		2.2 (0.4)	2.3 (0.1)		NS
P (mmol/l)		1.8 (0.6)	1.1 (0.2)		NS
Parathormone (pmol/l)		3.5 (1.4)	3.8 (1.4)		NS

Ca^2+^, total calcium; P, phosphorus. The values are presented as average (standard deviation).

*Significant (*p* < 0.05); NS, not significant.

In the correlation analysis of all variables studied, only the initial 25OHD level correlated with the final 25OHD level (SPRAY *p* = 0.021, r^2^ = 12.1%; GTTS *p* < 0.001, r^2^ = 25.4%). No association with dose was found. This may be explained by the low variability in the doses of vitamin D used, given the high compliance of the subjects. Therefore, it was not possible to create a valid regression equation or predict the required dose of vitamin D to maintain normal 25OHD levels.

## 4 Discussion

Vitamin D is a non-polar lipid. Its absorption is a complex, multi-step process. It begins with the decomposition of food in the stomach. After emulsification by bile acids in the small intestine, vitD micelles are absorbed by enterocytes through passive diffusion. This process is facilitated by several proteins (13). Additionally, the presence of another transporter in the jejunum is also assumed (13). The bioavailability of vitamin D is determined by multiple factors, of which only some are known. It depends on the pH of gastric juice, the activity of enzymes involved in fat digestion, the presence of bile acids, and potential interference by certain dietary micronutrients (vitamins A, E, K, long-chain fatty acids, and phytosterols) (14). This corresponds to the highly variable bioavailability of vitD among individuals (15).

In addition, the final plasma level of vitD is determined not only by intestinal absorption, but also by other factors such as vitamin D-binding protein, nuclear vitamin D-binding protein, and liver enzyme activity involved in vitD metabolism. Their genetic variability may significantly influence individual responses to supplementation (16).

Early studies suggested that IBD is one of the factors associated with vitD malabsorption (17). However the availability of vitD has only been studied in Crohn’s disease in remission (5). In these individuals, the increase in vitD levels following a single dose of vitD was approximately one-third lower than in healthy individuals. No correlation was found between the location of the disease and the degree of vitD absorption (5).

Given the poorly predictable absorption of vitamin D from the gastrointestinal tract (GIT), an attractive alternative to achieve reliable bioavailability is the use of formulations bypassing the GIT. A novel formulation based on vitD nanoemulsion has been developed. Nanomicelles pass through mucosal membranes more effectively. This leads to higher absorption rates after buccal and sublingual administration (18). Another advantage of oral absorption is the elimination of the first-pass effect through the liver (19).

Satia et al. (10) compared equivalent doses of vitamin D (1000 IU/day) administered either as a buccal spray or a soft gelatin capsule. The increase in 25OHD in the spray group was approximately twice as much (8 ng/ml) compared to the capsules. However, other studies have yielded inconsistent results. Todd at al. (7) did not confirm the higher efficacy of the oral spray (dose 3000 IU/day). Williams et al. (20), using the same dose and after 6 weeks of administration, also did not find any difference between capsules and spray (25OHD increase from 0.69 to 3.93 vs. 0.64–3.34 nmol/l/day). Two additional studies evaluated the effectiveness of supplementation in children or adolescents. In the first study (21), no difference was found. In the second one, Unsur et al. (22) reported higher levels of 25OHD after using the spray. In all cases, the studies included subjects without gastrointestinal diseases.

The only work by Satia et al. (10) included patients with IBD. Overall, there were 9 subjects with ulcerative colitis and 4 patients with Crohn’s disease, without further specification and separate evaluation.

To our knowledge, this is the first study to evaluate the efficacy of buccal nanoemulsion vitamin D supplementation in a well-characterized population of IBD patients. The increase in 25OHD levels we observed after conventional vitD (2000 IU/day) is approximately half of what Satia et al. reported (9 nmol/l vs. 20 nmol/l). However, this does not imply impaired vitD absorption in IBD. A similar increase (6 nmol/l) was documented after conventional vitD using the same dose (2000 IU/day) in healthy individuals (23).

We did not find any correlation between the change in 25OHD levels and other factors, such as the location of the disease or intestinal resection. This is not, given the sites of vitD absorption, a surprising finding. Similar conclusions have been reached by other authors (5, 16).

The variation in 25OHD levels after supplementation was similar for both the oral spray and conventional vitamin D. We, therefore, believe that, contrary to the original presumption, differences in intestinal absorption do not account for the variability in 25OHD levels. Other extraintestinal factors, such as those related to genetic polymorphisms in vitD metabolism, must have a predominant impact (14, 16, 24, 25). The initial level of 25OHD was the only factor influencing the change of 25OHD levels in our study, with an inverse relationship observed. This finding is consistent with prior studies, as several authors described a similar relationship (26, 27). We assume this is a pharmacokinetic consequence of the fact that 25-hydroxylase is a saturable enzyme. The production rate of 25OHD is directly proportional to the concentration of the substrate up to a limiting level of approximately 15 nmol/l (28).

The essential clinical question is the determination of the appropriate supplementation dose of vitD for IBD patients to maintain normal level. Both the National Academy of Medicine and the European Food Safety Authority recommend a daily vitamin D intake of 600 IU for healthy individuals up to 70 years of age (29, 30). However, several expert opinions have considered this dose insufficient and suggest a daily intake of 1500–2000 IU for adults (31, 32). Official guidelines for patients with inflammatory bowel disease (IBD) are lacking, except for specific conditions such as corticosteroid therapy (33).

None of the conducted studies have attempted to quantify the required dose of the orally vitD absorbable form. A reliable estimation of the required vitamin D dose could not be derived from our dataset. In patients with IBD, a daily dose of over 2000 IU of conventional oil-based vitamin D is required to maintain stable serum levels above 75 nmol/L (9). In our study, a dose of 1142 IU/day of an orally absorbable spray formulation, equivalent to half the conventional dose, proved equally effective. Based on these findings, we estimate that the required dose of the orally absorbable form is approximately 1000 IU/day. The comparable increase in 25(OH)D levels supports our hypothesis of improved bioavailability of the spray formulation.

### 4.1 Safety issues

Subjects did not report any side effects. Levels of Ca, P, and 25OHD did not exceed normal values. This vitD dosing is safe.

### 4.2 Study limitations

The vitamin D dose used was based on patient reports and was not objectively verified. The anticipated number of subjects could not be included in the analyses. Vitamin D was administered once weekly, and the daily dose was interpolated from the total weekly dose. The serum half-life of 25OHD is significantly longer than the dosing interval used. No difference in 25OHD levels was observed between daily and weekly dosing (34). Therefore, we believe this conversion is valid.

## 5 Conclusion

In patients with IBD, the buccal spray formulation of cholecalciferol achieved comparable efficacy to the conventional oil-based form, despite being administered at nearly half the dose (1143 IU/day vs. 2000 IU/day). This finding supports its superior bioavailability. Importantly, there is no risk of overdose at this dosage. Variable intestinal absorption is unlikely to explain inter-individual differences in 25OHD levels when using a conventional vitamin D emulsion.

## Data Availability

The datasets presented in this study can be found in online repositories. The names of the repository/repositories and accession number(s) can be found in the article/supplementary material.
